# The varying clinical effectiveness of single, three and five intraarticular injections of platelet-rich plasma in knee osteoarthritis

**DOI:** 10.1186/s13018-024-04736-6

**Published:** 2024-05-08

**Authors:** Weisheng Zhuang, Tianshu Li, Yuefang Li, Ying Zhang, Jiahuan Gao, Xu Wang, Qixin Ding, Wanyue Li

**Affiliations:** 1 Department of Rehabilitation Medicine, Henan Provincial People’s Hospital, People’s Hospital of Henan University, People’s Hospital of Zhengzhou University, Zhengzhou, Henan 450003 China; 2 Department of Rehabilitation, The First People’s Hospital of Zhengzhou, Zhengzhou, Henan 450003 China; 3 School of Rehabilitation Medicine, Henan University of Chinese Medicine, Zhengzhou, 450003 China; 4 Department of Rehabilitation, The First Affiliated Hospital of Jinan University, Guangzhou, Guangdong 510000 China

**Keywords:** Knee osteoarthritis, Platelet-rich plasma, Number of injections, Clinical effectiveness, Ultrasound

## Abstract

**Objective:**

To investigate the variations in clinical effectiveness among patients diagnosed with knee osteoarthritis who underwent intra-articular administration of platelet-rich plasma using single, triple, or quintuple injections.

**Methods:**

One hundred twenty patients with grade I-III knee osteoarthritis were randomly assigned to three groups: PRP1 group, who received a single injection of platelet-rich plasma; PRP3 group, who received three PRP injections one week apart; PRP5 group, who received five PRP injections one week apart. The patients’ conditions were evaluated using the Visual Analogue Scale (VAS) and the Western Ontario and McMaster Universities Arthritis Index-VA3.1 version (WOMAC-VA3.1) at baseline and 6, 12, 24, and 52 weeks 52 weeks follow up.

**Results:**

Out of the total participants, 106 patients (30 males and 76 females) completed the study. The primary outcome measure, WOMAC pain score, registered significant improvements across all groups when compared to pre-treatment levels. However, the application of 3 and 5 injections of platelet-rich plasma was substantially more effective than that of a single injection in reducing knee pain and stiffness, as well as enhancing physical function in patients with knee osteoarthritis. No statistically discernable difference was observed between PRP3 and PRP5 at all follow-up intervals, and there was no discernable difference between 3 and 5 PRP injections either. Mild side effects occurred in all three groups.

**Conclusions:**

The administration of three or five injections of platelet-rich plasma is safe, substantially more effective than single injections, and leads to remarkable clinical improvement by significantly reducing knee pain, improving joint stiffness, and enhancing physical function in patients with grade I-III knee osteoarthritis. Furthermore, no significant difference was observed in the efficacy of three or five injections. Therefore, we recommend using three injections of PRP in the treatment of patients with knee osteoarthritis of grade I-III.

**Supplementary Information:**

The online version contains supplementary material available at 10.1186/s13018-024-04736-6.

## Introduction

Knee osteoarthritis (KOA) is a degenerative pathology distinguished primarily by knee pain and functional impairment, arising from progressive deterioration of the articular cartilage due to multiple influence of metabolic, biomechanical, and biochemical factors. These factors lead to to an imbalance between anabolic and pro-inflammatory/catabolic processes, resulting in cartilage degradation. KOA is widespread among middle-aged and elderly population, with more than half of individuals aged 65 and above being afflicted. This condition has a significant economic impact on the family and society at large [1–4].

There is currently no cure for KOA, and no drugs or treatments are available to stop or reverse existing damage. Drug interventions can only temporarily relieve pain, while physical therapy, exercise, and other therapies primarily serve as adjunct measures with limited effectiveness. The current treatment strategy for KOA includes improving function, reducing disability, and alleviating pain, thereby enhancing the quality of life. While these approaches may slowing down the progression of structural and functional changes associated with KOA, they cannot completely cure KOA [5–7]. Platelet-rich plasma (PRP) therapy, which has regenerative and reparative effects on tissues, offers new hope for the treatment of KOA.

PRP is derived from autologous venous blood and, through centrifugal concentration, produces a concentrate with platelet counts exceeding the baseline blood levels. This concentrate is rich in high-concentration platelets, relevant growth factors, and other bioactive components. PRP may induce a regenerative response by enhancing the metabolic function of damaged structures and demonstrated a positive effect on cartilage formation and mesenchymal stem cell proliferation [8]. Moreover, PRP can also influence the inflammatory and angiogenesis processes, as well as the synthetic and breakdown metabolism balance in cartilage formation, and alter the existing microenvironment during disease development [9]. PRP therapy utilizes the biologically active regeneration cascade to stimulate natural healing responses and promote tissue healing. Orthopaedics and sport medicine doctors use PRP injections frequently for bone, ligament, and tendon injuries [10], as well as cartilage injuries and osteoarthritis [11–15]. Although PRP is a widely used minimally invasive treatment method in orthopedic and sports medicine fields to promote tissue healing and regeneration, there are still some unanswered questions remain, particularly regarding the optimal number of injections required for achieving the best clinical outcomes in treating KOA. Currently, no clinical guidelines or expert consensus on the optimal number of PRP injections required for the treatment of KOA.As a result, a consistent clinical standard for the optimal quantity of injections required for PRP treatment of KOA does not exist yet.

Despite the existing researchs, a consensus on the optimal number of PRP injections for the treatment of KOA remains elusive. Therefore, the aim of this study is to examine the clinical effects of varying numbers of PRP injections on KOA and establish the ideal number of injections that are necessary for successful treatment. Conducting such a study will enhance our comprehension of the effectiveness of PRP injections for KOA and provide valuable guidance for clinicians with respect to the number of injections they should administer to their patients. To achieve this objective, well-designed randomized controlled trials with appropriate sample sizes will be necessary. In this study, therefore, we aimed to compare the effectiveness of single, three and five doses of IA PRP in early stages of KOA.

## Materials and methods

### Study design and eligibility criteria

This study was a single-blind, three-arm, prospective, randomized, superiority trial (1:1:1 parallel allocation) study, and the study protocol was thoroughly reviewed and approved by the appropriate ethics committee (Ethical Lot Number: 2,018,057). Additionally, written, informed consent was obtained from all participating patients. The study protocol was registered with the China Clinical Trials Registry (Registration Date:09/02/2021, Clinical Registration Number: ChiCTR2100043259) to ensure transparency and accountability. We declare that all methods have been conducted in accordance with the relevant guidelines and regulations.

According to the previous study by Patel et al.(16), the change in the Visual Analog Scale (VAS) was chosen as the primary outcome measure for calculating the sample size. To achieve a study power of 80% (beta = 0.2) and a false-positive rate of 5% (alpha = 0.05), considering a predicted mean difference of 1.5 in VAS scores between treatment groups and a standard deviation of 0.6, it was determined that 35 patients per treatment arm would be required. To account for potential loss to follow-up, 40 patients were recruited per treatment arm, bringing the total sample size to 120 patients. Patients suffering from chronic (> 4 months) pain or swelling, along with grades 2 to 3 osteoarthropathy (graded according to the Kellgren Lawrence Tibiofemoral Joint Degeneration Scale) as determined by X-ray. Patients suffering from chronic (> 4 months) pain or swelling, along with grades 2–3 osteoarthropathy (according to the Kellgren Lawrence Tibiofemoral Joint Degeneration Scale) as determined by radiography at the Henan Provincial People’s Hospital between February 2021 and September 2022, were included. Conversely, the exclusion criteria included knee joint pain or swelling caused by rheumatoid arthritis, gout, serious cardiovascular disease, blood disease, malignant tumor, or infectious diseases. Immune impairment, anticoagulant treatment, nonsteroidal anti-inflammatory drug use within the past five days, hemoglobin count less than 11 g/dl, and platelet count less than 150,000/mm3 were also exclusion criteria.

**Fig. 1:**
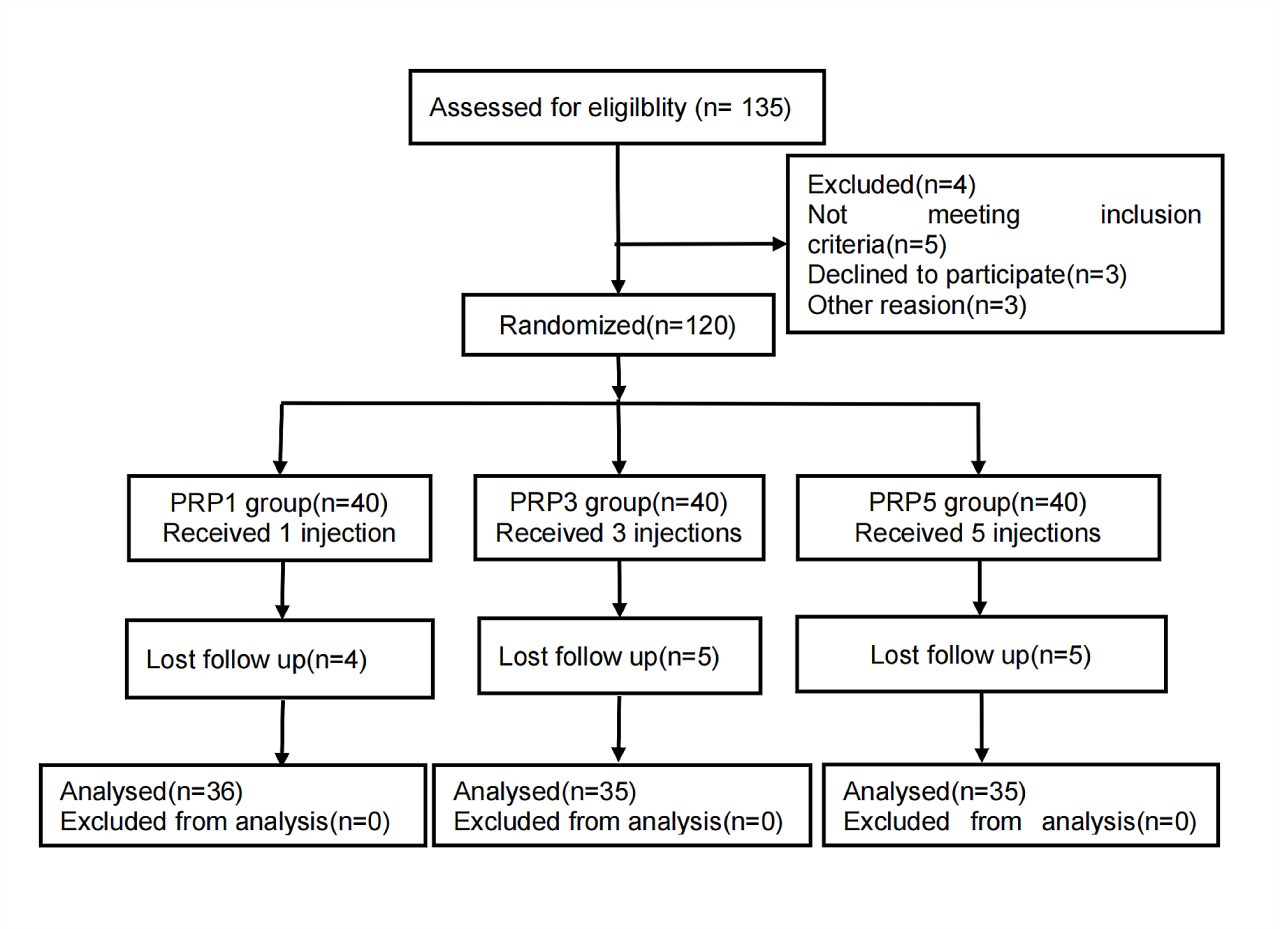
CONSORT flow diagram

Patients who met the inclusion criteria were classified into three groups, using a computer random method to ensure comparable baseline characteristics: PRP1, PRP3 and PRP5 group, with each group comprising 40 patients. The PRP1, PRP3, and PRP5 groups received one, three, and five PRP injections, respectively.The baseline characteristics included age, sex, course of disease, initial platelet concentration, and body mass index (BMI). The pain, stiffness, and physical function scores of the Western Ontario and McMaster Universities Osteoarthritis Index (WOMAC) subscales were measured at baseline and at weeks 6, 12, 24 and 52 weeks follow up. A flowchart of the study is shown in Fig. 1.

During the first visit, each patient underwent a preliminary assessment from a rehabilitation physician, and their medical history was recorded. Additionally, patients received a detailed instruction booklet and the WOMAC questionnaire, which they had to complete and bring on subsequent visits.

### Randomization and allocation concealment

The patients were allocated into three groups (PRP1, PRP3 and PRP5) through a randomization process conducted by an independent allocator. The process employed block randomization with block sizes varying randomly between two, four, and six, while maintaining concealed block sizes. The allocator generated random cards using computer-generated random numbers, which were subsequently placed in sealed envelopes. A duplicate set of sealed envelopes was created to address any potential need for code-breaking on an individual basis. During trial counseling and informed consent procedures, the sequentially numbered sealed envelope containing the patient’s allocation was opened and disclosed. As a result, the patients were categorized into the PRP1, PRP3 and PRP5 groups.

In the study, all injections were administered by a skilled rehabilitation attending physician. Both the patients and the attending physician in the rehabilitation department were unaware of the random grouping to minimize any potential bias. To ensure unbiased outcome evaluations, a dedicated observer who was oblivious of the group assignments completes all patient evaluations. Thereby maintaining objectivity and reducing any potential influence on the evaluation process. Additionally, the evaluators and the laboratory staff were kept blinded to the specific grouping of patients, further ensuring the integrity and impartiality of the study.

### PRP preparation and injection

PRP is obtained through a process of secondary centrifugation. Using aseptic techniques, a mixture of 2 ml of sodium citrate anticoagulant (product lot number: 190,228,266, Sichuan Nangal Biotechnology Co., Ltd.) and 18 mL of blood is extracted from the patient’s median elbow vein. This mixture is then subjected to low-speed centrifugation (model H1850, Hunan Xiangyi Centrifuge Instrument Co., Ltd.) with a first centrifugal force of 200 g during 10 min and a second centrifugal force of 200 g during 20 min. Following the initial centrifugation, the whole blood was separated into three layers. The supernatant, junctional layer, and a layer of red blood cells approximately 3 mm in thickness beneath the junctional layer were collected for the second centrifugation. Upon completion of the centrifugation process, approximately 3/4 of the upper plasma layer was removed, leaving behind approximately 4 mL of PRP. A sterile syringe with a capacity of 5 mL was used to extract the remaining portion. Blood and PRP samples were randomly and regularly collected from patients receiving treatment. Both types of samples were analyzed using a blood analyzer (Sysmex XN-9100, Sysmex Corporation, Japan) to verify the correct preparation of PRP and ensure compliance with manufacturer specifications. The platelet concentration in the PRP prepared using this method is approximately four times higher than the baseline level. The initial platelet concentration was 235.61 ± 38.47 × 10^9^/L. The average concentration of PRP obtained from blood samples was 928.57 ± 39.78 × 10^9^/L, which contained leukocytes and a small amount of red blood cells. According to the latest new classification and coding system pertaining to platelet-rich plasma for the management of KOA [16], the PRP codes in this study are 29-11-00. The PRP characteristics in this study are detailed in Table 1.

**Table 1:** Baseline Characteristics of Participant

Groups	N	Gender		Age	K-L classification			Initial platelet concentration	Platelet concentration in PRP	Leukocyte concentration in PRP	BMI (k/m2)
		male	female	(years, $\bar x + s$)	Grade I	Grade II	Grade III	(*10^9^/L, $\bar x + s$)	(*10^9^/L, $\bar x + s$)	(*10^9^/L, $\bar x + s$)	(k/m^2^, $\bar x + s$)
PRP1	36	11	25	58.75 ± 8.88	8	13	15	241.44 ± 31.98	969.44 ± 104.49	14.92 ± 3.04	25.63 ± 1.99
PRP3	35	9	26	59.88 ± 8.34	9	10	16	232.68 ± 28.02	965.74 ± 89.89	15.80 ± 3.25	26.01 ± 2.06
PRP5	35	10	25	59.54 ± 7.49	10	11	14	229.61 ± 28.76	959.80 ± 117.51	16.10 ± 2.96	26.37 ± 2.08
*χ* ^*2*^ */F*		0.207		0.177	0.029			1.178	2.346	1.718	1.157
*P*		0.902		0.838	0.971			0.313	0.098	0.182	0.318

No: number, BMI: body mass index. NOTE. Quantitative variables were expressed as mean standard deviation. Qualitative variables were shown as absolute and relative frequencies. *P* < 0.05 was considered statistically significant

**Fig. 2:**
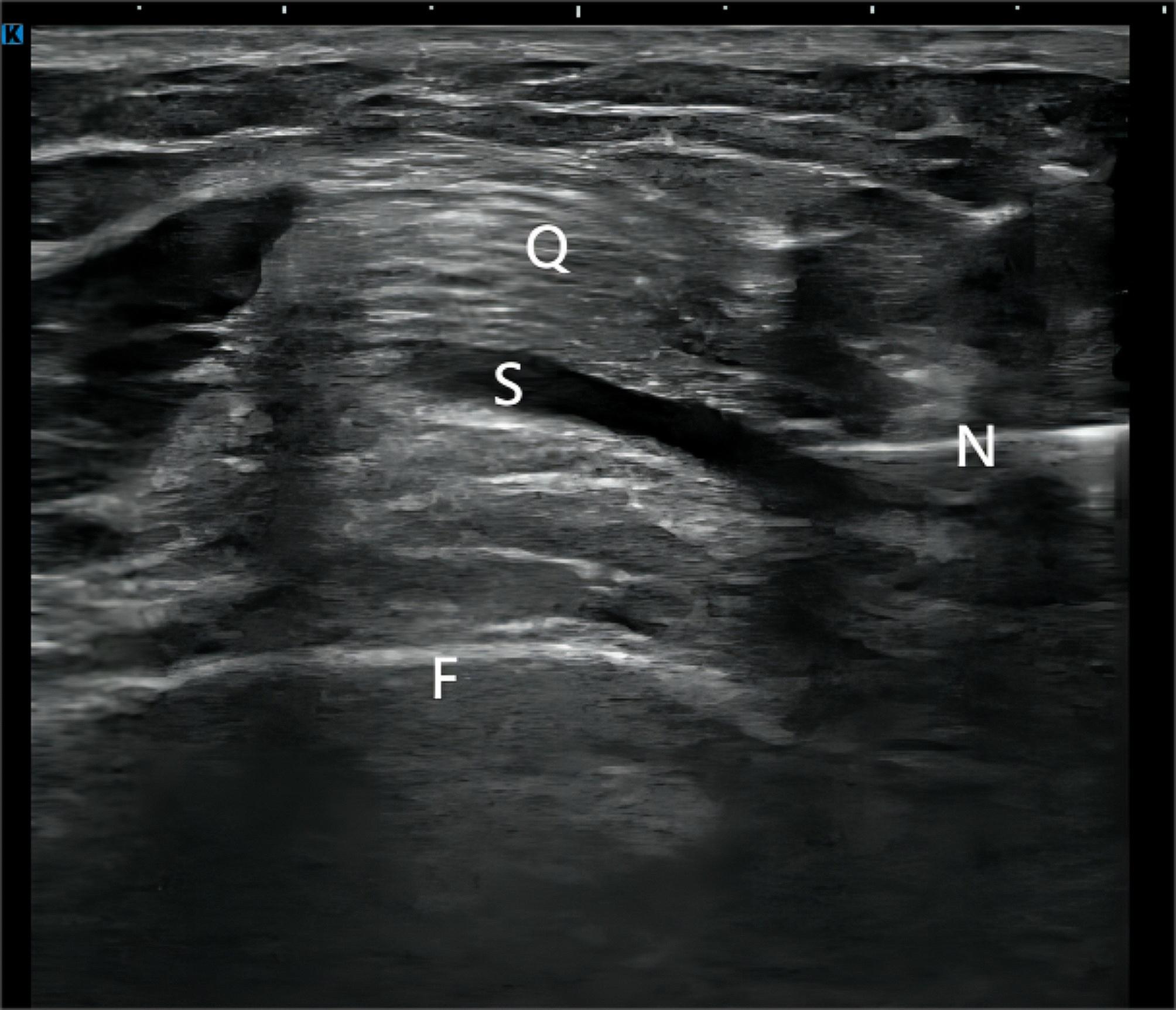
Platelet-rich plasma injection guided by ultrasound. F: Femur, Q:Quadriceps tendon, N:Needle, S:Suprapatellar bursa

The injection technique entailed the patient assuming a supine position while naturally extending both lower limbs, thereby exposing the entire injection site. Following the placement of sterile towels and the application of sterile coupling agents, 4mL of PRP was injected into the suprapatellar capsule under the guidance of Konica Minolta Co., Ltd.‘s Sonimage HS1 Plus model ultrasound system(Fig. 2). The same rehabilitating attending physician administered the PRP injections for all patients. After the injection is completed, patients were advised to rest for 30 min while being closely monitored for any potential adverse events. Additionally, patients were instructed to refrain from water contact at the injection site for 1 day and to avoid engaging in vigorous activities for a week. The different groups were established to evaluate the potential effects of varying numbers of PRP injections on the outcome measures being assessed in the study. In the study, the PRP injection treatment interval was one week. The patients in the PRP1 group received a total of one PRP injection, whereas the PRP3 group received three PRP injections over the course of three weeks. Similarly, the PRP5 group received a total of five PRP injections administered once a week for five consecutive weeks. Aspirin, acetaminophen, and non-selective non-steroidal anti-inflammatory drugs (NSAIDs) inhibit platelet aggregation and can reduce the effectiveness of PRP. Therefore, their use was avoided throughout the duration of this study [17]. Patients were solely permitted to use COX-2-selective NSAIDs and cold compress as analgesics [18]. All patients underwent comprehensive health education before the injection, encompassing consistent guidance on home-based exercise, moderate aerobic training, lifestyle modifications, adherence to a nourishing diet, weight management, refraining from engaging in mountain climbing and weightlifting endeavors, limitations on climbing stairs, avoidance of squatting and sitting cross-legged. All participants were strongly advised to strictly adhere to these recommendations.

### Outcome measures

The primary outcome involved the use of the Western Ontario and McMaster University Osteoarthritis Index Scale -VA3.1 version (WOMAC-VA3.1) to evaluate the knee joint function of patients, which mainly included the WOMAC total score, pain, stiffness, and function scores. The secondary outcomes comprised the VAS scores, changes in WOMAC total score and VAS scores at 6, 12, 24 an52 weeks follow up, and the percentage of decrease in these scores relative to baseline.

The assessment of the patients’ knee joint pain and function was conducted at baseline, 6, 12, 24 and 52 weeks follow up by the same rehabilitation therapist. This evaluation employed the WOMAC and VAS. Notably, the scale evaluation was performed by nonexperimental personnel.

### Data analysis

Statistical analyses were conducted using SPSS version 26.0 software (IBM Corp., Armonk, NY, USA). Descriptive data were expressed as the mean ± standard deviation (SD) or median (min, max) for continuous variables, and the frequency and percentage for categorical variables. Normality was assessed using the D’Agostino Pearson test. Where appropriate, chi-squared and Fisher’s exact tests were used to compare categorical variables between study groups. To assess the changes in WOMAC and VAS scores at different time intervals within each study group, repeated measures analysis of variance (ANOVA) was performed. Subsequently, the Bonferroni post-hoc test was used to examine pairwise comparisons between the time periods. One-way ANOVA and Tukey post-hoc test were applied to to compare the scores among the three study groups at each time interval. Moreover, the ANOVA was used to compare the average age, hemoglobin levels, white blood cell counts, and platelet count among the three study groups. The significance level was set at α = 0.05 (two-sided), with *P* < 0.05 indicating statistical significance.

## Results

### Patients characteristics

The study enlisted 120 eligible patients at the Henan Provincial People’s Hospital between February 2021 and September 2022. Of the initial patient cohort, four patients in the PRP1 group and five patients each in the PRP3 and PRP5 groups withdrew from the study. Consequently, a total of 106 patients successfully completed the study (refer to Fig. 1). The baseline characteristics of the three groups were comparable with respect to the patients age, sex, disease duration, initial platelet concentration, mean platelet, and white blood cell counts, and BMI. No discernible differences were observed in the clinical demographics among the three groups (Table 2).

**Table 2:** Classification Features of PRP

Parameter	Values
PRP preparation	
Initial blood volume	18 ml
Anticoagulant	2.5% sodium citrate 2 ml
System	Close
Centrifugation	Yes
Number	2
First centrifugal force	200×*g* for 10 min
Second centrifugal force	200×*g* for 20 min
Final PRP volume	4 ml
PRP characteristics	
PRP type	29-11-00
Initial blood platelet concentration	235.61 ± 38.47(10^9^/L)
Platelet concentration in PRP	962.57 ± 39.78(10^9^/L)
Does it contain red blood cells? (0 = No, 1 = Yes)	1
Does it contain white blood cells? (0 = No, 1 = Yes)	1
Initial leukocyte concentration	5.36 ± 1.63(10^9^/L)
Leukocyte concentration in PRP	15.80 ± 3.25(10^9^/L)
Mode of activation(0 = Endogenous activation, 1 = Activation prior to injection.)	0
Calcium agent used for activation(0 = No, 1 = Yes)	0
Application characteristics	
Formulation type	Liquid
Administration route	Intraarticular
Dosage	A treatment session is conducted once a week
Volume	Intraarticular injection: 4 ml
Dose (range of platelets)	Intraarticular injection:3.67 × 109–4.03 × 10^9^
Tissue	Cartilage, synovium, subchondral bone
Pathology	Knee joint degeneration

### Clinical results

The means, standard deviations, P-values, and 95% confidence intervals of the three groups were compared, revealing significant mean differences between PRP1 and PRP3, as well as PRP1 and PRP5 (*P* < 0.001). From Table 3, No statistically significant differences (*p* > 0.05) were observed in the WOMAC total, pain, stiffness, and function scores, as well as the VAS score at baseline among the patients in the PRP1, PRP3, and PRP5 groups. However, significant differences were observed in the WOMAC and VAS scores among the PRP1, RP3, and PRP5 groups at the 6, 12, 24, and 52-week follow up post-intervention (*P* < 0.001). The scores across all three groups were the highest at baseline and decreased post-intervention, with the PRP1 group reaching the lowest score at 6 weeks follow up and gradually increase thereafter until the 52-week mark. The PRP3 and PRP5 groups reached their lowest scores at 12-week follow up, followed by a gradual increase, peaking at 52 weeks. Despite a statistical difference (*P* > 0.05) in WOMAC and VAS scores for the PRP1 group between 52 weeks post-intervention and at baseline, the difference was deemed clinically insignificant due to its small magnitude. In comparison to the baseline, significant differences were noted in WOMAC and VAS scores for the PRP3 and PRP5 groups at 6, 12, 24, and 52-week follow up (*P* < 0.05). Additionally, no statistically significant difference was observed in the WOMAC and VAS scores between the PRP3 and PRP5 groups at 6, 12, 24, and 52-week follow up post-intervention (*P* > 0.05).

**Table 3:** Comparison of WOMAC and VAS scores at each follow-up stage baseline and post-treatment in the 3 groups

			Follow-up				^c^P Value
Treated Groups	Group	Baseline	6 weeks	12 weeks	24 weeks	52 weeks	
W.Total Mean (95%CI)	PRP1	68.67(65.01,72.32)	44.89(41.75,48.02) ^b^	47.28(43.77,50.69) ^b^	55.00(51.29,58.71) ^b^	61.03(57.13,64.93) ^b^	0.001
	PRP3	68.69(64.34,73.03)	33.31(30.52,36.11) ^a^	25.89(23.89,27.88) ^a^	28.89(26.52,31.25) ^a^	31.80(29.22,34.38) ^a^	< 0.001
	PRP5	69.40(65.18,73.72)	29.91(27.94,31.89) ^a^	23.40(22.09,24.71) ^a^	25.97(24.11,27.84) ^a^	28.17(26.01,30.33) ^a^	< 0.001
	P Value	0.958	< 0.001	< 0.001	< 0.001	< 0.001	
W.Pain	PRP1	15.11(14.57,15.65)	10.97(10.48,11.47) ^b^	11.61(11.07,12.16) ^b^	13.14(12.58,13.69) ^b^	14.14(13.61,14.66) ^b^	0.08
	PRP3	14.97(14.42,15.53)	7.14(6.60,7.68) ^a^	5.43(5.07,5.78) ^a^	6.00(5.66,6.34) ^a^	6.80(6.46,7.14) ^a^	< 0.001
	PRP5	15.29(14.77,15.80)	6.54(6.19,6.89) ^a^	5.06(4.76,5.36) ^a^	5.49(5.16,5.81) ^a^	6.23(5.86,6.60) ^a^	< 0.001
	P	0.706	< 0.001	< 0.001	< 0.001	< 0.001	
W.Stiffness	PRP1	4.39(4.08,4.69)	3.06(2.79,3.32) ^b^	3.17(2.87,3.36) ^b^	3.42(3.12,3.71) ^b^	3.92(3.61,4.22) ^b^	0.13
	PRP3	4.17(3.80,4.54)	2.31(2.04,2.59) ^a^	1.57(1.38,1.76) ^a^	1.69(1.47,1.90) ^a^	2.00(1.75,2.25) ^a^	< 0.001
	PRP5	4.14(3.77,4.52)	2.11(1.83,2.40) ^a^	1.43(1.19,1.67) ^a^	1.49(1.22,1.75) ^a^	1.80(1.50,2.10) ^a^	< 0.001
	P	0.542	< 0.001	< 0.001	< 0.001	0.007	
W.Function	PRP1	49.17(46.05,52.28)	30.86(27.96,33.76) ^b^	32.50(29.46,35.54) ^b^	38.44(35.21,41.68) ^b^	42.97(39.49,46.44) ^b^	0.002
	PRP3	49.54(45.86,53.23)	23.86(21.35,26.37) ^a^	18.89(17.17,20.61) ^a^	21.20(19.03,23.37) ^a^	23.00(20.51,25.49) ^a^	< 0.001
	PRP5	49.98(46.31,53.64)	21.26(19.45,23.07) ^a^	16.91(15.71,18.12) ^a^	19.00(17.24,20.76) ^a^	20.14(18.16,22.13) ^a^	< 0.001
	P	0.946	0.001	< 0.001	< 0.001	< 0.001	
VAS	PRP1	7.81(7.49,8.12)	5.72(5.43,6.01) ^b^	6.03(5.74,6.31) ^b^	6.81(6.53,7.08) ^b^	7.36(7.08,7.64) ^b^	0.015
	PRP3	7.60(7.19,8.01)	4.57(4.06,5.09) ^a^	4.40(3.72,5.08) ^a^	5.11(4.40,5.83) ^a^	5.71(4.93,6.49) ^a^	< 0.001
	PRP5	7.54(7.16,7.93)	4.23(3.68,4.78) ^a^	3.86(3.14,4.57) ^a^	4.34(3.59,5.09) ^a^	4.66(3.92,5.39) ^a^	< 0.001
	P	0.562	0.001	< 0.001	< 0.001	< 0.001	

W: WOMAC. a-statistically significant differences between groups at each time point compared with the PRP1 group(*P* < 0.05), b Significant difference from PRP3、PRP5 group. c*p*-Significant difference from baseline within each respective group(*P* < 0.05). SD: standard deviation

**Fig. 3:**
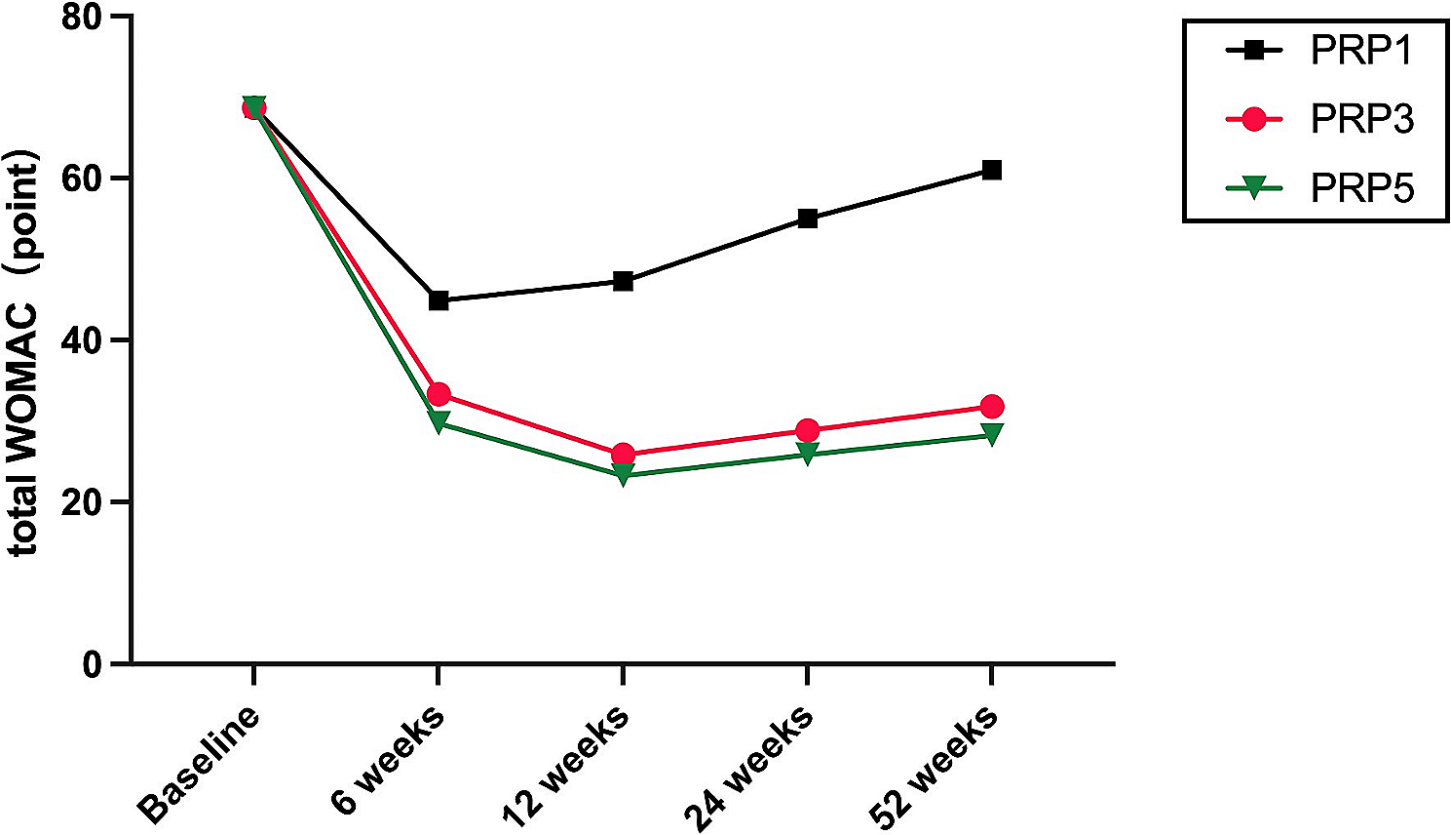
the changes of WOMAC total score variables from baseline to 6,12,24 and 52 weeks

**Fig. 4:**
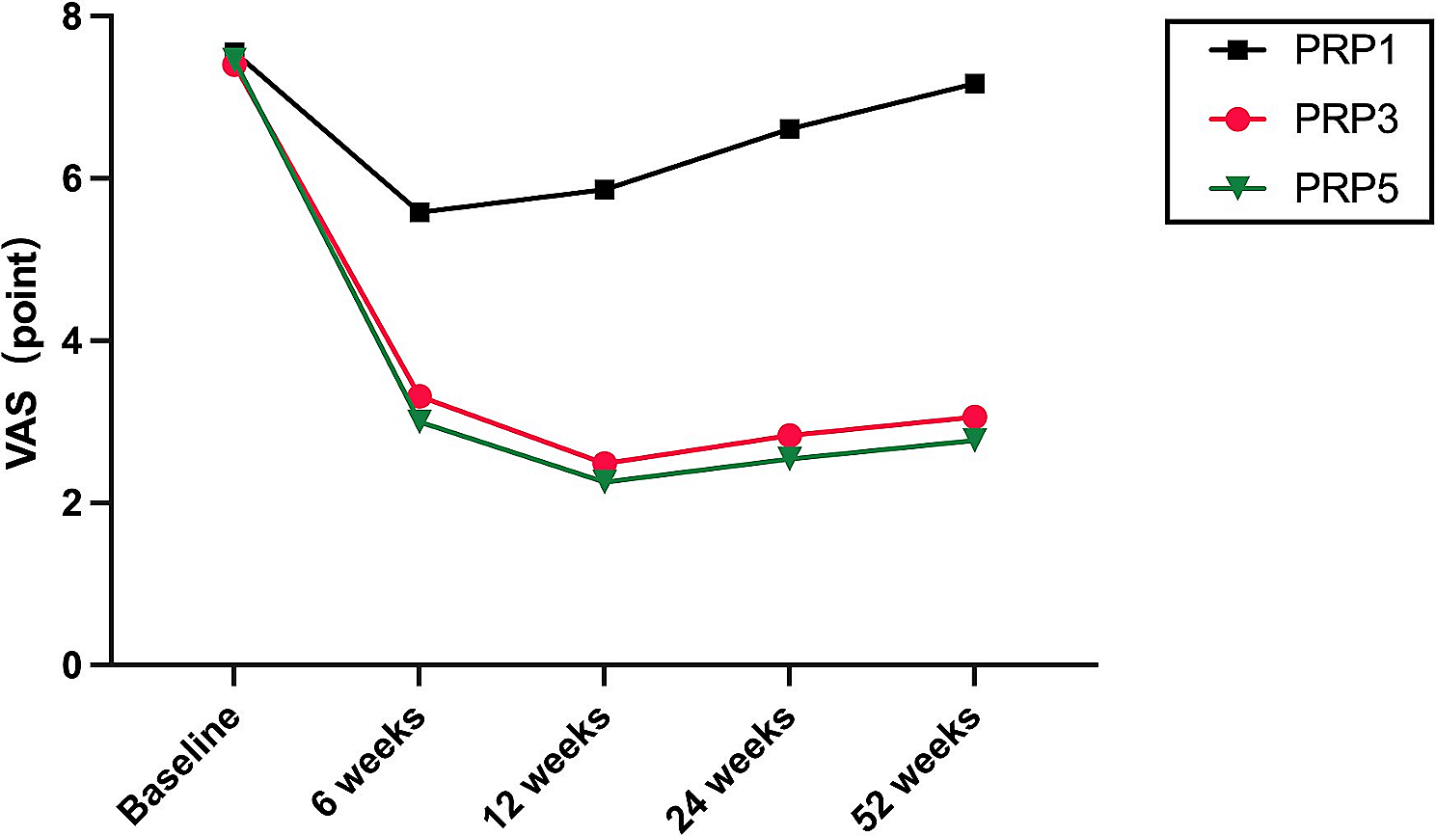
the changes of VAS score variables from baseline to 6,12,24 and 54 weeks post-treatment

Figures 3 and 4 illustrate the trajectory of WOMAC total and VAS scores from baseline to weeks 6, 12, 24,and 52 following the treatment.It can be observed that patients in the PRP3 and PRP5 groups experienced a decline in WOMAC and VAS scores at 6 and 12 weeks post-intervention. Notably, the decline was more pronounced in the PRP5 group, with a gradual increase observed after 24 weeks. Conversely, the PRP1 group exhibited an upward trend in WOMAC total and VAS scores starting from 6 weeks, reaching its peak at 52 weeks. The increase was more significant in the PRP1 group (*P* < 0.01). Overall, the WOMAC and VAS scores of all three groups increased after 24 weeks follow up, with the maximum increase observed at 52 weeks.

**Fig. 5:**
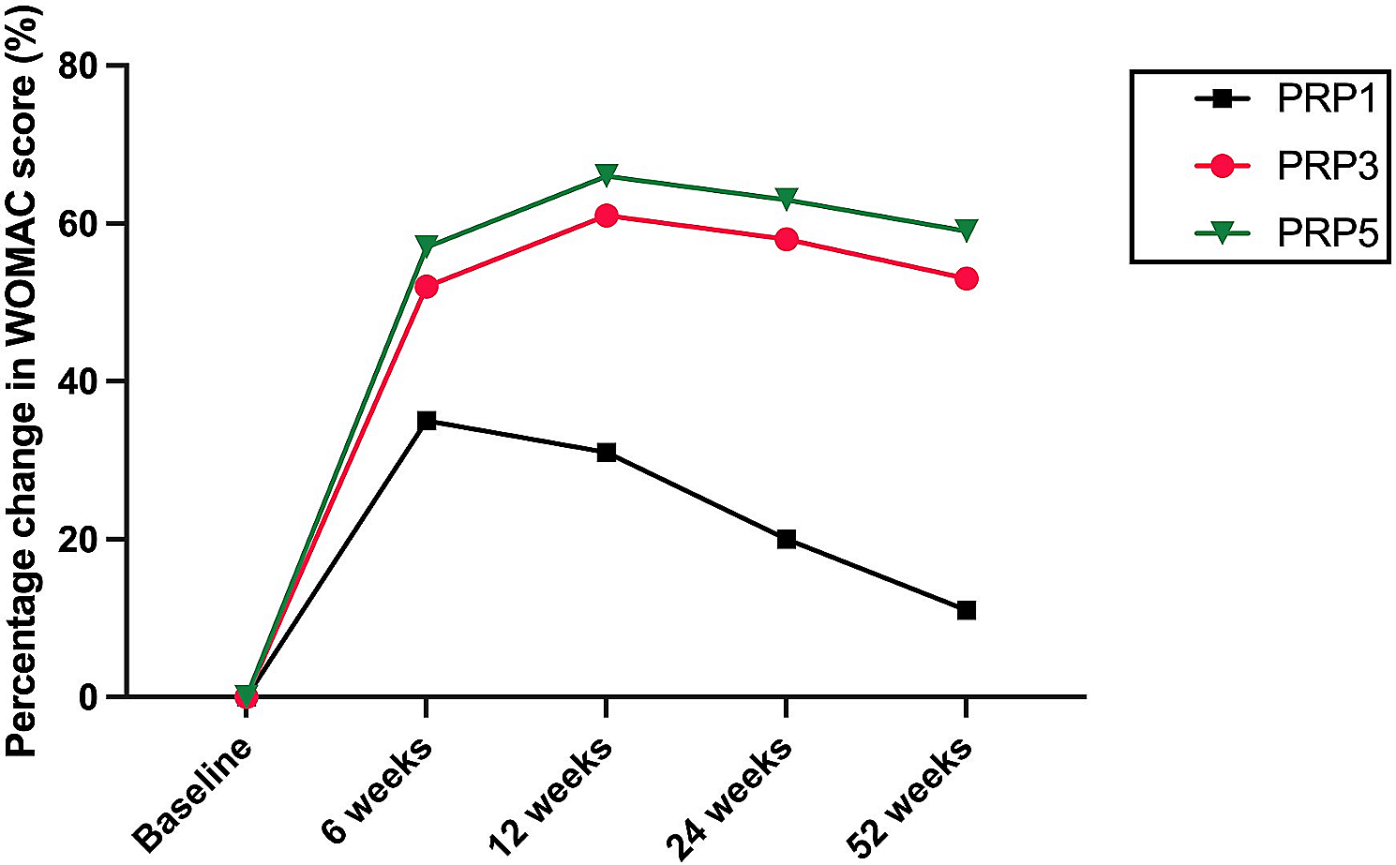
the percentage decrease of WOMAC relative to baseline

**Fig. 6:**
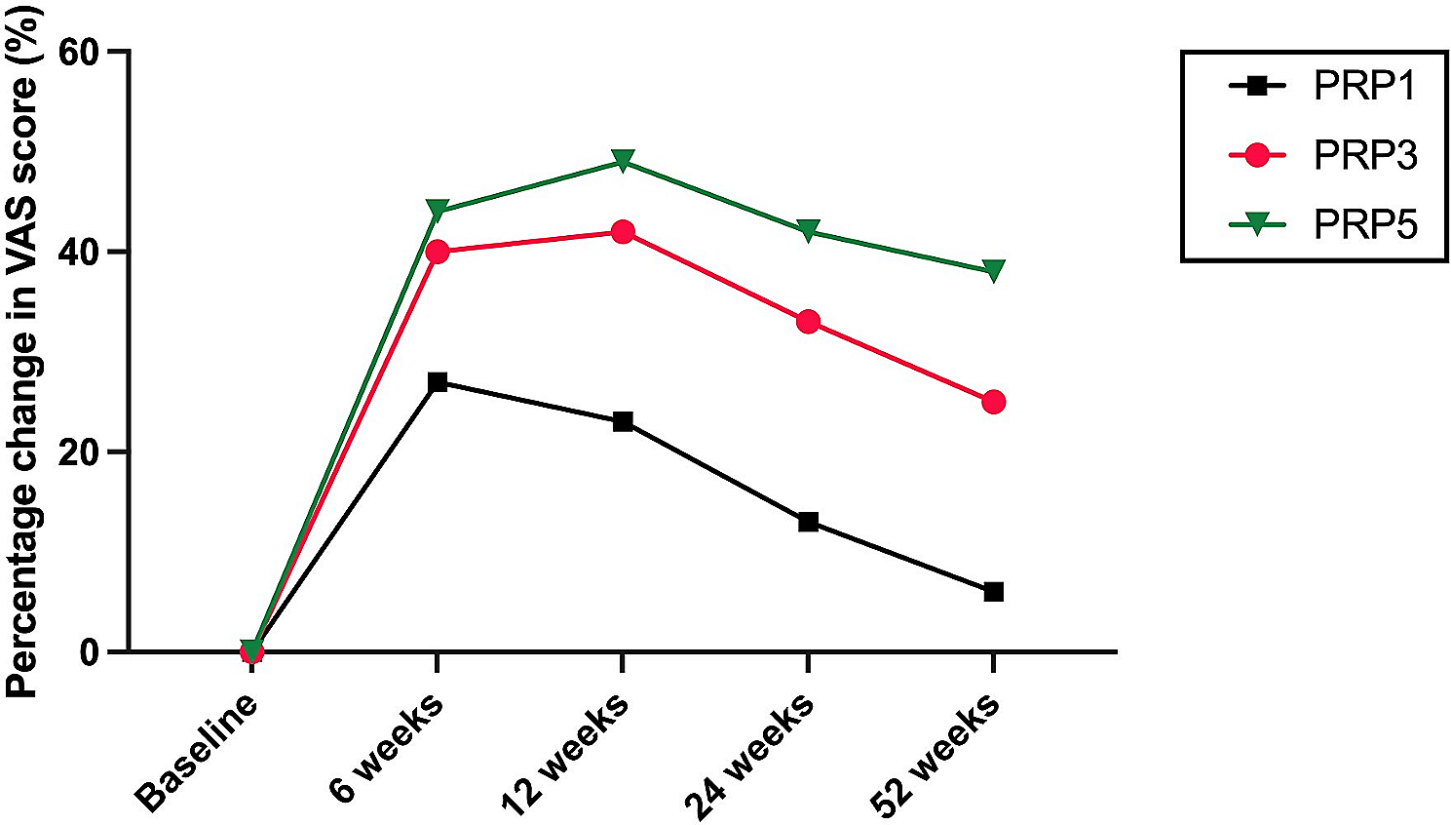
the percentage decrease of VAS relative to baseline

Figures 5 and 6 show the percentage decrease of VAS and WOMAC score relative to baseline over the course of 6 to 54 weeks follow up. At 6, 12, 24, and 52 weeks follow up, both PRP3 and PRP5 groups demonstrated significant percentage improvement compared to the baseline in PRP3. Significant improvement was observed between PRP5 and PRP1 groups (*P* < 0.05) but not between the PRP3 and PRP5 groups. The percentage improvement compared to baseline in the PRP3 and PRP5 groups peaked at 12 weeks follow up and gradually declined after 24 weeks, reaching its lowest point at 52 weeks. This suggests that the efficacy of PRP treatment is most optimal within the initial 24 weeks, progressively diminishing thereafter, with the least efficacy observed at 52 weeks. Importantly, there were no statistically significant differences in the VAS score compared to the baseline in the PRP1 group at 52 weeks(*P* = 0.05).

### Adverse effects

PRP therapy for KOA is generally considered safe as it utilizes the patient’s own blood components. However, some potential adverse effects should be be considered, such as infection, post-injection pain or discomfort, injection site bleeding, and allergic reactions. During treatment and follow-up, no significant complications were observed, except for a temporary increase in local pain or swelling. The main adverse reaction of PRP therapy reported in this study were pain in the injection site and discomfort, comprising 16 occurrences. Among them, 3 occurrences were in the PRP1 group, 5 occurrences were in the PRP3 group, and 8 occurrences were in the PRP5 group. The pain symptoms were relatively mild, and patients reported relief of pain within 48 h through bed rest or local cold compress. No adverse events such as injection site infection or allergic reactions occurred.

## Discussion

While numerous clinical studies examined the use of PRP for treating KOA at various injection frequencies, considerable variations exists in PRP preparation methods, such as inclusion of white blood cells in PRP, injection techniques, dosage, use of activators and local anesthetics, and other factors. Moreover, different studies employ varying evaluation methods for assessing efficacy and have diverse follow-up periods. Consequently, no current definitive consensus exists on the ideal treatment frequency for intra-articular PRP injections to effectively manage KOA.

Currently, 9 clinical studies investigated the effects of different injection regimens on the efficacy of PRP treatment forKOA (Table 4). Of these studies, eight compared the efficacy of multiple versus single PRP injection, while one study compared the efficacy of two against four injections. All of these studies concluded that PRP injections are effective in treating KOA, resulting in significant pain reduction and improvement in knee joint function [19–27]. One study found no statistically significant difference in efficacy between single and double injections [19], as well as between single and triple injections [22]. Six studies reported that multiple injections were more effective than single injections [19–27]. In one study comparing the efficacy of single and double injections, double injections were found to be superior [24]. Five studies compared the efficacy of single and triple injections and concluded that triple injections were more effective. Two studies suggested that triple injections were more effective than both single and double injections [22], with one study recommending a minimum of two injections. One study compared the clinical efficacy and levels of inflammatory factors in synovial fluid between two and four injections for the treatment of KOA. Both doses significantly improved the clinical symptoms of patients with KOA, with no statistical difference between the two groups. However, neither regimen had an impact on the levels of inflammatory factors, such as interleukin-6, interleukin-1β, and TNF-α n the synovial fluid.However, the use of PRP activation and application of activators vary among the studies.In five studies, calcium chloride was used to activate PRP, while one study utilized 10% calcium gluconate for activation. Three studies did not use any activator to activate PRP. Most of the studies did not administer local anesthesia at the injection site before PRP injection, except for two studies that used lidocaine for local anesthesia prior to injection. Six studies did not classify the PRP preparations used. One study classified them as P2Bb according to the Platelets, Activation, White cells (PAW) classification, and two studies classified them as 2 A and 4B according to the Mishra classification. Additionally, the volume of PRP per dose used in each study also varies, with most studies using a volume of 4-5mL and the maximum volume being 8mL. The time intervals between the two PRP injections also varied significantly. Two studies had a one-week interval, three studies had a two-week interval, three studies had a six-week interval, and one study had a one-month interval. The follow-up duration also differed among the studies with a minimum follow-up of three months and a maximum follow-up of two years. Four studies had a six-month follow-up, while three studies had a one-year follow-up. The aforementioned 9 clinical studies exhibit substantial variations in research methods, inclusion criteria, PRP preparation methods, types of PRP, presence of white blood cells, injection methods, PRP activation and activators, injection doses, use of local anesthetics, follow-up period, efficacy evaluation methods, and other aspects. Consequently, their findings diverge significantly and even result in contradictory conclusions (Table 4). In addition, the effectiveness of PRP treatment for KOA is not only influenced by the frequency of treatment, but also by the quality of the PRP, which is determined by factors such as patient age, immune status, presence of concurrent metabolic diseases, and medications used [28].

**Table 4:** Comparison of randomized trials on different injections and dose of platelet rich plasma in osteoarthritis of knee, as available in literature

NO	Study	Year	Inclusion criteria	Leukocyte Concentrations	PRP classification	Sample size(No. of knees analyzed)	PRP activation	Volume of PRP per dose	Anesthesia	Duration between repeat injections	Duration of follow-up	Conclusion
1	Patel et al.7	2013	Ahlback Grade 1, 2	LP-PRP	Mishra4B	Placebo-46 1-dose PRP-52 2-dose PRP-50	10% calcium chloride	8 mL	No	3 weeks	6 months	1 and 2 dose groups comparable
2	Görmeli et al.8	2014	All KL Grades	LR-PRP	No mention	Placebo-40 Hyaluronic acid-39 1-dose PRP-44 3-dose PRP-39	10% calcium chloride	5 mL	No	1 week	6 months	3-doses superior to 1-dose PRP
3	Kavadar et al.11	2015	KL Grade 3	LR-PRP	No mention	1-dose PRP-33 2-dose PRP-32 3-dose PRP-33	10% calcium chloride	4–5 mL	No	2 weeks	6 months	3 doses superior to 2 dose, superior to 1 dose. At least 2 doses recommended.
4	Usulu Güvendi et al.9	2017	KL Grade 3	LR-PRP	No mention	Corticosteriod-19 1-dose PRP-19 3-dose PRP-19	No activation	No mention	No	1 week	6 months	1 and 3 dose groups comparable
5	Simental-Mendía et al.10	2019	KL Grade 1, 2	LP-PRP	No mention	1-dose PRP-18 3-dose PRP-17	10%calcium gluconate solution	5 mL	2%lidocaine Local anesthesia	2 weeks	1 year	3 doses superior to 1 dose
6	Tavassoli et al.12	2019	Ahlback Grade 1, 2	No mention	No mention	Hyaluronic acid-62 1-dose PRP-62 2-dose PRP-66	No activation	4–6 mL	No	3 weeks	3 months	2 doses superior to 1 dose
7	Subramanyam K et al.13	2021	KL Grade 1, 2	LP-PRP	PAW P2Bb	1-dose PRP-33 2-dose PRP-32 3-dose PRP-33	No activation	4 ml	topical anesthesia	2 weeks	1 year	3 doses superior to 1 、2dose PRP
8	Ngarmukos et al.	2021	KL Grade 1, 2, 3, 4	LP-PRP	No mention	2-dose PRP-51 4-dose PRP-43	10% calcium chloride	5-7 ml	No	6 weeks	1 year	2and 4 dose groups comparable
9	Yurtbay et al.	2021	KL Grade 1, 2, 3	LR-PRP	Mishra 2 A	1-dose Placebo-59 3-dose Placebo-53 1-dose PRP-62 2-dose PRP-63	10% calcium chloride	5 ml	No	1month	2 year	3-doses superior to 1-dose PRP

PRP: Platelet-rich plasma; LP-PRP: leukocyte-poor PRP; LR-PRP: leukocyte-rich PRP; KL: Kellgren-Lawre; PAW: Platelets, Activation, White cells classification

Based on these studies, three intra-articular PRP injections can yield favorable clinical outcomes for knee joints. However, we hypothesize that additional injections may be necessary to achieve even better clinical results. For instance, undergoing a treatment consisting of five injections might lead to improved outcomes. Therefore, we compared the effectiveness between groups receiving three and five injections. Considering the insights derived from these studies, we adopted a standardized approach in preparing PRP, ensuring consistency in the composition of its various components. Depending on the concentration of white blood cells, it can be divided into leukocyte-poor and leukocyte-rich PRP. The leukocyte-rich PRP used in this study has a better anti-inflammatory effect [29]. Research has shown that local anesthetics may have a toxic effect on knee joint cartilage cells and can affect platelet activation by altering the intra-articular pH. Therefore, local anesthetics were not used prior to PRP injection [30]. Based on the substantial clinical evidence supporting the efficacy of PRP in treating KOA, we excluded a placebo control group or other injection interventions, such as steroids or hyaluronic acid,, as comparators. We selected patients with bilateral KOA at KL 1–3 stages as our study population, with simultaneous injection and functional assessment conducted on both knees of each patient. Building upon previous research that primarily compared the effects of three PRP injections with single or two PRP injections, we investigated the differences in efficacy between single, three and five PRP injections. Each injection consist of 4mL of PRP per joint, with a one-week interval between injections.

Ultrasound-guided injection was demonstrated to be more precise and accurate compared to blind injections in existing literature [31]. Therefore, we employed ultrasound guidance for intra-articular PRP injections, which was one of the contributing factors for achieving improved therapeutic outcomes in this study.

The results of this study demonstrate that, compared to baseline, the VAS scores and WOMAC index of all three groups improved at 6 weeks, 12 weeks, 24 weeks and 52 weeks after intervention. This indicates that intra-articular PRP injections significantly enhance the function and alleviate joint pain in patients with KOA, which is consistent with the findings of previous studies. However, at the same time points after intervention, both the VAS and WOMAC scores of the PRP3 and PRP5 groups were statistically superior to those of the PRP1 group (*P* < 0.05). This suggests that multiple injections have better clinical efficacy than a single injection, which aligns with the conclusions of previous studies. Görmeli G et al. [20] discovered that three injections of PRP showed superior efficacy compared to single injections for patients with early KOA. Similarly, Kavadar G et al. [21] suggested that two PRP injections had a better prognosis than a single injection. Mehdi et al. [23] found that two PRP injections were more effective than a single injection and both were more effective than hyaluronic acid injections, which aligns with our findings. A most recent meta-analysis incorporating seven clinical studies involving 575 patients suggests that triple-dose PRP therapy may be more effective than single-dose PRP in the treatment of KOA [32]. Additionally, the VAS and WOMAC scores showed a decreasing trend after 6 months of PRP intervention, which became more pronounced after one year of intervention. Although this result suggests that multiple are more effective than single injections in the treatment of KOA, the principle of “more is better” does not necessarily apply. The results of this study revealed no significant difference (*P* > 0.05) in VAS and WOMAC scores between the PRP3 and PRP5 groups at the same time points following PRP intervention. Our research findings primarily suggest that three injections provide the maximum benefit to patients and may serve as a potential option for delaying KOA surgery or as substitute surgical treatment.

The possible mechanism for the better effect of multiple injections compared to a single injection in this study could be attributed to the growth factors in PRP that continue to promote cartilage and tissue metabolism with a cumulative effect. In vitro experiments on PRP therapy for KOA have shown significant dose and time dependence in terms of the quantity and metabolic activity of chondrocytes [33]. Moreover, a clinical study indicates that PRP therapy has a dose-dependent cumulative effect. In this study, multiple injections of PRP combined with PRF (Platelet Rich Fibrin) were administered, with a maximum of four injections. This treatment approach could alleviate pain, improve knee joint function, function, and delay the need for knee replacement surgery, with the clinical effects becoming more evident with a higher number of treatment sessions [34].

However, there was no significant difference in clinical efficacy between the 3 and 5 injections, and the reasons for this result are remain unclear. Nevertheless, this finding is consistent with many clinical studies that recommend a three-injection treatment protocol. These findings align with that of Ngarmukos et al. (25) where they observed that despite dosage variations between the group receiving two and four injections, both groups demonstrated improvement in clinical scores without any statistically significant difference in clinical efficacy.

PRP contains a variety of growth factors and cytokines, such as platelet-derived growth factor (PDGF), transforming growth factor-beta (TGF-β), and vascular endothelial growth factor (VEGF), which promote cell proliferation, differentiation, and tissue repair. PRP is extensively used in clinical practice for conditions such as tendinopathies and cartilage injuries. The mechanism of action behind PRP injections for KOA lies in its high concentrations of platelets and growth factors, including PDGF, TGF-β1, basic fibroblast growth factor (bFGF), and VEGF. These substances can stimulate the production of fibroblasts and collagen, regulate the joint microenvironment, enhance cell viability [35, 36] promote angiogenesis, and facilitate myogenic cell proliferation [37]. KOA is characterized by the degeneration of articular cartilage, local synovial inflammation, joint capsule contracture, and ligamentous laxity or contracture [38]. The synergistic effects of growth factors present in PRP can promote cartilage regeneration and metabolism, potentially exerting a chondrogenic effect.

In KOA, which is characterized by synovial inflammation, cartilage erosion, and infiltration of inflammatory cells, PRP treatment can work through several mechanisms: (1) First, it promotes tissue repair and regeneration. The growth factors and cytokines in PRP stimulate the proliferation and synthesis of chondrocytes, promoting the repair and regeneration of damaged tissues [39, 40]. (2) Second, PRP exhibits anti-inflammatory properties. The growth factors in PRP also possess anti-inflammatory effects, reducing the infiltration of inflammatory cells and alleviating associated inflammatory reactions. This helps to relieve the symptoms of arthritis [41]. (3) Third, PRP improved joint lubrication and function. The introduction of PRP into affected joints can enhance the viscoelasticity and lubricating properties of synovial fluid, leading to improved joint mobility and function [42]. (4) Finally, PRP therapy offers analgesic effects. PRP therapy can potentially relieve pain symptoms by reducing inflammation and promoting tissue repair. These mechanisms collectively contribute to the effectiveness of PRP in the treatment of KOA [41]. Additionally, neovascularization facilitated by PRP can nourish contracted muscles and tissues, thereby slowing down the progression of KOA. These effects can function independently or even interact additively and synergistically [37].

This study has certain limitations. While implementing patient blinding would have bolstered the validity of our findings, it would have necessitated the administration of placebo injections, a course of action deemed ethically inappropriate. Furthermore, this study is constrained by its exclusive reliance on a single clinical research center and a relatively small sample size. To ascertain the optimal frequency of PRP treatment for KOA, more robust clinical evidence is still warranted. Conducting large-scale, multicenter clinical trials utilizing randomized double-blind controlled methodologies, extending the follow-up duration, assessing treatment efficacy comprehensively, and observing the long-term effects of PRP are imperative. Additional fundamental research is indispensable in elucidating the precise mechanisms underlying the therapeutic use of PRP in the treatment of KOA.

## Conclusion

In conclusion, this study provides evidence that, when it comes to treating knee osteoarthritis with PRP therapy, multiple injections (3 and 5) result in superior clinical outcomes compared to a single injection. However, no significant difference was observed in the efficacy of three or five injections. Therefore, we recommend using three injections of PRP in the treatment of patients with knee osteoarthritis of grade I-III.

### Electronic supplementary material

Below is the link to the electronic supplementary material.

Supplementary Material 1

Supplementary Material 2

## Data Availability

No datasets were generated or analysed during the current study.
