# Supplementary material for: The varying clinical effectiveness of single, three and five intraarticular injections of platelet-rich plasma in knee osteoarthritis
Source: J Orthop Surg Res. 2024 May 8;19:284. doi: 10.1186/s13018-024-04736-6 (PMC11077828; doi:10.1186/s13018-024-04736-6)
Supplement: Supplementary file 2 — Supplementary Material 2 [file 13018_2024_4736_MOESM2_ESM.docx]

Dear Reviewer

We sincerely appreciate you for your patient and meticulous review of our manuscript. We are grateful for the detailed and constructive suggestions provided. As English is not our native language, resulting in numerous grammar and writing errors in the manuscript. We have diligently revised these errors and sought the assistance of native English speakers for proofreading and editing. Following each reviewer's suggestions, we have made revisions to the manuscript and highlighted these changes in red within the manuscript.

We have responded to the reviewer's comments one by one .

1. remove comma between ‘’three’’ and ‘’five’’

We have removed the comma, as detailed in the manuscript.

3,4,6,7,9 add a space after the comma when citing the author's name and postal code

19 add a space after colon

22 add a space after colon

We have added spaces after the commas in lines 3, 4, 6, 7, 9, 19, and 22, as indicated in the manuscript.

23-25 I think that it is more suitable to say ‘’ who received three (five) prp injections one week apart’’

Following your advice, we have revised the content in lines 23-25 to read "who received three (five) PRP injections one week apart," and highlighted this in red in the manuscript.

28 it is better to use ‘’…52 weeks follow up’’ than ‘’post-treatment’’.

Following your suggestion, we have replaced all instances of "post-treatment" with "follow up" in the manuscript and marked them in red.

29 add a space after colon

We have added spaces after the comma in line 29

32 it is better to use ‘’single injection’’ instead of ‘’singular’’

Following your suggestion, we have replaced "singular" with "single injection" and marked it in red in the manuscript.

34-35 it is better to use ‘’difference’’ instead of ‘’variance’’, ‘’significant/discernable.

Following your suggestion, we have replaced "variance" and "significant/discernable" with "difference" and marked it in red in the manuscript.

36 it is better to use ‘’ mild side effects occurred in all three groups’’.

Following your suggestion, we have included the phrase "mild side effects occurred in all three groups" in the manuscript and highlighted it in red.

37 add a space after colon

We have added spaces after colon in line 37

38 again is better to use ‘’single’’ instead of ‘’singular’’

Following your suggestion, we have replaced "singular" with "single injection" and marked it in red in the manuscript.

40-42 it is better to say ‘’ no significant difference was observed in the efficacy of three or five injections. Therefore, we recommend using three injections of PRP in the treatment of patients….’’

Based on your recommendation, we have made revisions and included the sentence "no significant difference was observed in the efficacy of three or five injections. Therefore, we recommend using three injections of PRP in the treatment of patients" in the manuscript, highlighted in red.

47 it better to use ‘’ multiple influence of…’’

Following your advice, we have incorporated the phrase "multiple influence of" in the manuscript and highlighted it in red.

49 it is better to use ‘’elderly population’’

Following your advice, we have incorporated the phrase "elderly population" in the manuscript and highlighted it in red.

50 it is better to use ‘’ this condition has a significant economic impact on the family and society’’

Following your suggestion, we have included the phrase " this condition has a significant economic impact on the family and society" in the manuscript and highlighted it in red.

52 it is better to use ‘’there is currently no cure for KOA

Following your advice, we have incorporated the phrase "there is currently no cure for KOA" in the manuscript and highlighted it in red.

53 it is better to use just ‘’exercise’’ instead of ‘’exercise therapy’’

Following your suggestion, we have replaced "exercise therapy" with "exercise" and marked it in red in the manuscript.

55-56 it is better to use ‘’slowing down the progression’’, and ‘’healing’’ instead of ‘’cure’’

Following your suggestion, we have replaced "cure" with "slowing down the progression" and marked it in red in the manuscript.

60 it is better to say ‘’Orthopaedics and sport medicine doctors are use PRP injections frequently for…

Following your advice, we have incorporated the phrase "Orthopaedics and sport medicine doctors are use PRP injections frequently for…" in the manuscript and highlighted it in red.

65, 66 add a space after dot

We have added spaces after dot

69 it is better to use ‘’single injection’’

Following your advice, we have incorporated the phrase "single injection" in the manuscript and highlighted it in red. Due to modifications and adjustments made to this section, it has been moved to the discussion later on.

75 add a space after Lawrence

We have added spaces after Lawrence. Due to modifications and adjustments made to this section, it has been moved to the discussion later on.

81 add a space (for≤2)

We have added spaces (for≤2)

82 it is better to use : ‘’not exist yet’’

Following your advice, we have incorporated the phrase "not exist yet" in the manuscript and highlighted it in red.

84 plural : researchs

We have made modifications to the “researchs”, but it has been in line 75

90-91 add a space after dot, remove comma in ‘’three, and five’’

Following your advice, we have made the modifications.

100, 106, 109 add a space after dot

Following your advice, we have made the modifications.

113 which radiology methods were used? X-ray, MRI, CT?

X-ray were used,marked it in red in the manuscript.

114 gout is enough

Following your advice, we have made the modifications and highlighted it in red.

115 anticoagulant are doubled

Thank you for your attention to detail, we have made the modifications.

117 11g/dl, not dL

Thank you for your attention to detail, we have made the modifications.

119, 120 add a space after comma, separate ‘’methodto’’

Thank you for your attention to detail, we have made the modifications.

122 add a space after dot

Thank you, we have made the modifications.

125 it is better to use : WERE MEASURED AT BASELINE AND AT 6, 12, 24 AND 52 WEEKS FOLLOW UP

Following your advice, we have incorporated the phrase "WERE MEASURED AT BASELINE AND AT 6, 12, 24 AND 52 WEEKS FOLLOW UP" in the manuscript and highlighted it in red.

151- 152 consider DURING 10 MINUTES, instead of FOR A DURATION OF

Following your suggestion, we have replaced "DURING 10 MINUTES" with "FOR A DURATION OF" and marked it in red in the manuscript.

157 add a space after dot Thank you, we have made the modifications.

165 add a space after dot Thank you, we have made the modifications.

175, 176 add a space after dot Thank you, we have made the modifications.

177 interval WAS not IS

Thank you for your attention to detail, we have made the modifications and highlighted it in red.

182 add a space after dot

Thank you, we have made the modifications.

185 it is better to use CLIMBING than ASCENDING

Following your advice, we have incorporated the phrase "CLIMBING" in the manuscript and highlighted it in red.

191 add a space after comma, separate WOMACtotal

Thank you, we have made the modifications.

193 add a space after comma, it is better to use FOLLOW UP than POST-INTERVENTION

Thank you for your attention to detail, we have made the modifications and highlighted it in red.

194 separate SCORESRELATIVE

Thank you, we have made the modifications.

196 it is better to use FOLLOW UP than AFTER THE INTERVENTION

Thank you for your attention to detail, we have made the modifications and highlighted it in red.

201 separate: min,max

Thank you, we have made the modifications.

209 add a space after comma Thank you, we have made the modifications.

240 better is FOLLOW UP than POST TREATMENT; ad a space after comma and dot.

Thank you for your attention to detail, we have made the modifications and highlighted it in red.

252 separate PRPtherapy

Thank you, we have made the modifications.

255, 256, 258 add a space after comma and dot. It is better were FOUND in the….

Thank you, we have made the modifications.

262 add a space after dot

Thank you, we have made the modifications.

271 separate : osteoarthritis(Table4)

Thank you, we have made the modifications.

275 -282 separate words from brackets, add a space after dot and comma

Thank you, we have made the modifications.

296 – 327 add a space after comma and dot, separate the words from brackets

Thank you, we have made the modifications.

325 injection WAS consist

Thank you, we have made the modifications.

342 separate RECENTMETA

Thank you, we have made the modifications.

344 – 380 add a space after dot, separate words from brackets

Thank you, we have made the modifications.

350 no need, BECAUSE, just THE RESULTS OF…

Following your advice, We have removed "Because."

371 no need BETWEEN THE TWO

Following your advice, We have removed "BETWEEN THE TWO"

381 – 409 add a space after comma and dot

Thank you, we have made the modifications

381 what does it mean: DEGENERTIVE DEGENERTION?

Thank you,this is due to our carelessness and lack of proficiency in English writing. We have made the modifications and marked them in red in the document.

386 when enumerating, put numbers : 1) Tissue repair and regeneration: ….2) Anti-

inflammatory….3)…

Thank you,we have put numbers, and highlighted it in red.

415 separate THATTHERE

Thank you, we have made the modifications

Regarding the tables and figures, the remarks are as follows:

In Table 1. in the year column you missed letter S for YEAR; font is not unique; add a space between words and punctuation in table explanation.

Thank you, we have made the modifications

In figures you don’t need to write WEEKS LATER, just WEEKS

Following your advice, We have removed "LATER"

In ‘’DECLARATION’’ separate THATHERE; use the same font

Thank you, we have made the modifications

In ‘’HIGHLIGHTS’’ it is better to use MOST SUITABLE or MORE SUITABLE than one or five injections.

Following your advice, we have incorporated the phrase "MOST SUITABLE" in the ’HIGHLIGHTS and highlighted it in red.

We want to express our gratitude once again for your thorough, meticulous, and patient review, as well as for providing the best revision suggestions. Instead of rejecting our paper outright due to our poor English writing skills, you offered detailed recommendations for improvement, for which we are truly thankful. We send our warmest wishes to you and wish you everlasting health and happiness.

Dr Zhuang

Henan Provincial People’s Hospital

2024.3.20
