# Supplementary material for: The varying clinical effectiveness of single, three and five intraarticular injections of platelet-rich plasma in knee osteoarthritis
Source: J Orthop Surg Res. 2024 May 8;19:284. doi: 10.1186/s13018-024-04736-6 (PMC11077828; doi:10.1186/s13018-024-04736-6)
Supplement: Supplementary file 1 — Supplementary Material 1 [file 13018_2024_4736_MOESM1_ESM.docx]

Dear editor/sir/madam

We sincerely appreciate the editor and reviewers for their patient and meticulous review of our manuscript. We are grateful for the detailed and constructive suggestions provided. As English is not our native language, we lack proficiency in academic English writing, resulting in numerous grammar and expression errors in the manuscript. We have diligently revised these errors and sought the assistance of native English speakers for proofreading and editing. Following each reviewer's suggestions, we have made revisions to the manuscript and highlighted these changes in red within the manuscript.

Dear reviewers

Thank you very much for your review and the suggested revisions.We have made revisions to address each question raised by the reviewers, providing detailed explanations and responses to their queries. These changes have been marked in green within the revision notes.

1.Introduction Revision: Ensure that the introduction is informative and sets the stage for hypothesis development. Move lines 65-81 to the Discussion section and revise the Introduction accordingly to maintain coherence.

Thank you for your valuable suggestions. Following your advice, we have revised the introduction section accordingly. Additionally, we have incorporated lines 65-81 into the discussion section seamlessly.

2.Methodology Section: Remove unnecessary citation in line 99. In my opinion, the rest of the Methodology section is clear and reproducible.

Based on your advice, the citation from line 99 has been removed.

3.Results Section: Rephrase the statement in line 223 regarding the reference to Table 3 for analysis. Make it clearer how Table 3 is referenced. Consider rephrasing to ensure clear indication of where Table 3 is referenced.

In accordance with your advice, we have rephrased the content pertaining to Table 3 in line 223 of the Results section and provided a more detailed description of its contents. This section has been highlighted in red in the revised manuscript.

4.Discussion section: Table 4 and Comparison: Question the necessity of Table 4 and the comparison with other studies. Consider if a descriptive comparison within the section suffices. Other parts of the Discussion section seem fair.

First of all, although there are numerous studies on PRP therapy for knee osteoarthritis (KOA), there is a scarcity of research comparing the efficacy of PRP with different injection frequencies. Currently, only the studies listed in Table 4 can be retrieved. However, due to the multitude of factors influencing the efficacy of PRP, such as variations in PRP concentration, platelet count, PRP dosage, PRP classification, presence of leukocytes, age of subjects, injection methods, activation status, concurrent medications, immune status, metabolic diseases, etc., the heterogeneity between studies is significant. This has led to contradictory results among different studies. Therefore, the detailed description in Table 4 and the comparison between studies are primarily intended to provide readers with a more intuitive display of the differences between these studies and the potential factors causing these differences, serving as a reference for future similar research and aiding clinical decision-making.

Furthermore, this study is single-centered, which introduces a small sample bias and limitations in terms of stability and reliability of the data, potentially influenced by individual events or factors. By comparing our results with those of other studies, mutual corroboration can enhance the persuasiveness and credibility of the findings, providing a basis for clinical physicians when considering PRP as a treatment strategy for KOA.

Thirdly, Table 3 provides a detailed description of the specific research methods and differences in results among the studies, offering a more detailed portrayal of the current status and existing issues in PRP treatment for KOA. This indicates a lack of international standardized norms in PRP research, leading to varying research outcomes and reduced credibility. Therefore, the establishment of international and standardized PRP standards and research methods is urgently needed, which can encourage future researchers to adopt internationally recognized norms and methods in conducting PRP research.

In summary, this manuscript extensively lists and describes previous studies comparing the efficacy of different PRP injection frequencies in Table 4, which may seem redundant but is indeed essential. If the reviewers consider this section redundant, we are open to considering its removal.

Thanks again, I wish you all the best in the further
